# Gut microbiota contributions to anorexia nervosa pathogenesis: insights from the activity-based anorexia model

**DOI:** 10.1038/s41522-026-01055-y

**Published:** 2026-06-23

**Authors:** Radka Roubalová, Janet Luthar, Petra Procházková, Kateřina Zadáková, Kristýna Coufalová, Jakub Kreisinger, Jaroslav Semerád, Alena Nehasilová, Hynek Mácha, Dominika Luptáková, Helena Tlaskalová-Hogenová, Petra Holanová, Alena Lambertová, Hana Papežová

**Affiliations:** 1 Institute of Microbiology of the Czech Academy of Sciences, Prague, Czech Republic; 2 First Faculty of Medicine, Charles University, Prague, Czech Republic; 3 Department of Zoology, Faculty of Science, Charles University, Prague, Czech Republic; 4 Institute for Environmental Studies, Faculty of Science, Charles University, Prague, Czech Republic; 5 Department of Psychiatry, First Faculty of Medicine, Charles University and General University Hospital in Prague, Prague, Czech Republic

**Keywords:** Diseases, Microbiology, Neuroscience

## Abstract

Anorexia nervosa (AN) is a severe eating disorder that profoundly affects quality of life. Despite increasing understanding of the neurobiological basis of the disease, many patients develop a chronic course of illness accompanied by a variety of physical and psychiatric comorbidities. To investigate the contribution of the gut microbiome to disease progression, we employed the activity-based anorexia (ABA) mouse model of AN. We performed fecal microbiota transplantation using samples from three donor groups: healthy controls, patients with acute AN, and patients with severe and enduring AN (SEAN). We continuously assessed changes in the gut microbiota and fecal metabolites (e.g., short-chain fatty acids, serotonin, and GABA) throughout the experiment, along with behavioral traits across the three groups of mice. Mice colonized with microbiota from acute AN patients exhibited reduced hunger signaling (via NPY, AgRP, MCH, and orexin), accompanied by decreased food intake. In contrast, mice transplanted with microbiota from SEAN patients showed appetite signaling and food consumption comparable to those colonized with microbiota from healthy controls but displayed significantly higher running activity relative to the other groups. However, the distinct microbiota did not affect the development of the ABA phenotype. Overall, our findings suggest that changes in the gut microbiome during disease development contribute to disease progression. Our results also indicate that restoration of a healthy gut microbiome is essential for complete recovery.

## Introduction

Anorexia nervosa (AN) is a severe eating disorder with an etiology that is not yet well understood. Due to very low caloric intake and increased physical activity, patients experience severe psychological and physical health comorbidities. A major concern is the relatively low rate of full recovery and the high proportion of patients who develop chronic AN^1^. In addition to the chronicity of the disease, the increased mortality risk in patients with AN highlights the need to explore new therapeutic strategies and treatments.

Over the past decade, the gut microbiome has gained increasing attention in relation to human behavior and neuropsychiatric disorders^2^. The various mechanisms by which microbiota can modulate signaling along the gut–brain axis have been recently described^3^. These include autonomic and enteric nervous signaling, endocrine effects of hormones and neurotransmitters produced in the gastrointestinal tract, and products of the gut mucosal immune response^4^. The gut microbiome is a source of compounds such as microbe-associated molecular patterns (MAMPs), short-chain fatty acids (SCFAs), amino acid derivatives, and secondary bile acids that can act through specific receptors on intestinal epithelial cells, immune cells, and neurons to modulate gut–brain signaling^3^. Some of these substances are produced by both microorganisms and the host and are essential in microbial endocrinology. Typically, these include neuroactive molecules such as serotonin, γ-aminobutyric acid (GABA), and catecholamines, which can affect bacterial growth and gene expression^5^. In patients with AN, altered levels of these neurotransmitters in both serum and feces have been reported^6^, which may disrupt the bi-directional communication between the microbiome and the host and contribute to the development of AN.

Diet is the key modifiable factor shaping microbiome composition and functional potential^7^. The microbiota and its metabolic products, in turn, influence the production of gastrointestinal tract hormones such as glucagon-like peptide-1 (GLP-1), cholecystokinin (CCK), and peptide YY (PYY), peripheral appetite regulators that promote satiety and reduce food intake^8^. In anorexia nervosa, impaired homeostatic control of food intake and energy expenditure has been observed at the level of both peripheral and central peptides that regulate eating behavior^9^. At the same time, dysbiosis of the gut microbiome has been observed in several studies of patients with AN^10^. Research into the connection between microbiome dysbiosis and impaired appetite regulation in the pathophysiology of anorexia nervosa is therefore of great importance.

In our most recent study, we focused on the differences in microbiome and metabolome profiles between patients suffering from acute AN and those with SEAN^11^. Our results showed significant differences in gut microbiota diversity and composition not only between AN patients and healthy controls, but also between acute and chronic patients. Additionally, we observed significant alterations in SCFA levels and subtle differences in fecal neurotransmitter levels between healthy controls and AN patients. To determine whether these changes merely reflect physiological and metabolic adaptations to AN or also contribute to disease chronicity, we employed a mouse model of anorexia nervosa. We transplanted gut microbiota from healthy controls, acute AN patients, and SEAN patients into mice and applied the activity-based anorexia (ABA) model, a well-established mouse model used for decades to study the neurobiological basis of AN^12^. The ABA model combines food restriction with access to a running wheel, resulting in weight loss and hyperactivity, which are core features of human AN. We hypothesized that the core features of AN would develop differently in “AN-like” mice colonized with distinct microbiota and that these differences would be linked to specific gut microbiota signatures affecting signaling along the gut–brain axis.

## Methods

### Participants

Ten female patients with acute early-stage disease (acute AN; less than three years of disease duration), ten female patients with chronic severe and enduring AN (SEAN; more than seven years of disease duration), and ten healthy female controls (HC) were included in our study. These patients were selected from our larger cohort, which we described recently^11^. Healthy individuals and patients with acute AN were randomly selected. To effectively distinguish the effect of microbiota from acute and chronic patients, we preferentially selected SEAN patients with a longer duration of AN (median disease duration of 198 months in the present cohort compared to 102 months in the original cohort). We determined the physical and clinical parameters and medication use of the participants (Table S1). Blood samples were taken from the cubital vein of all participants early in the morning in the Center for Eating Disorders of the Psychiatric Department of the First Faculty of Medicine of Charles University and at the General University Hospital in Prague. Stool samples were collected from all participants, immediately processed (*Preparation of fecal microbiota for fecal microbiota transplantation* section), and frozen at −80 °C. Some stool samples were fresh-frozen at −80 °C to observe the effect of processing on microbial profile (Fig. S1). Participants were asked not to drink alcohol, coffee, or black tea, not to eat any products containing cocoa, chocolate, nuts, or bananas and not to take probiotics for two days before stool collection.

The exclusion criteria for study enrollment included pregnancy, breastfeeding, severe active or chronic diseases, infectious diseases, and the use of antibiotics or antimycotics within the three months prior to participation. Healthy subjects had no history of eating disorders or other psychiatric illnesses. Additionally, there was no evidence of a genetic predisposition to eating disorders in family histories.

The study was conducted in accordance with the Declaration of Helsinki and was approved by the Ethics Committee of the General University Hospital, Prague, Czech Republic. Written informed consent was obtained from all participants.

### Preparation of fecal microbiota for fecal microbiota transplantation

**Fig. 1:**
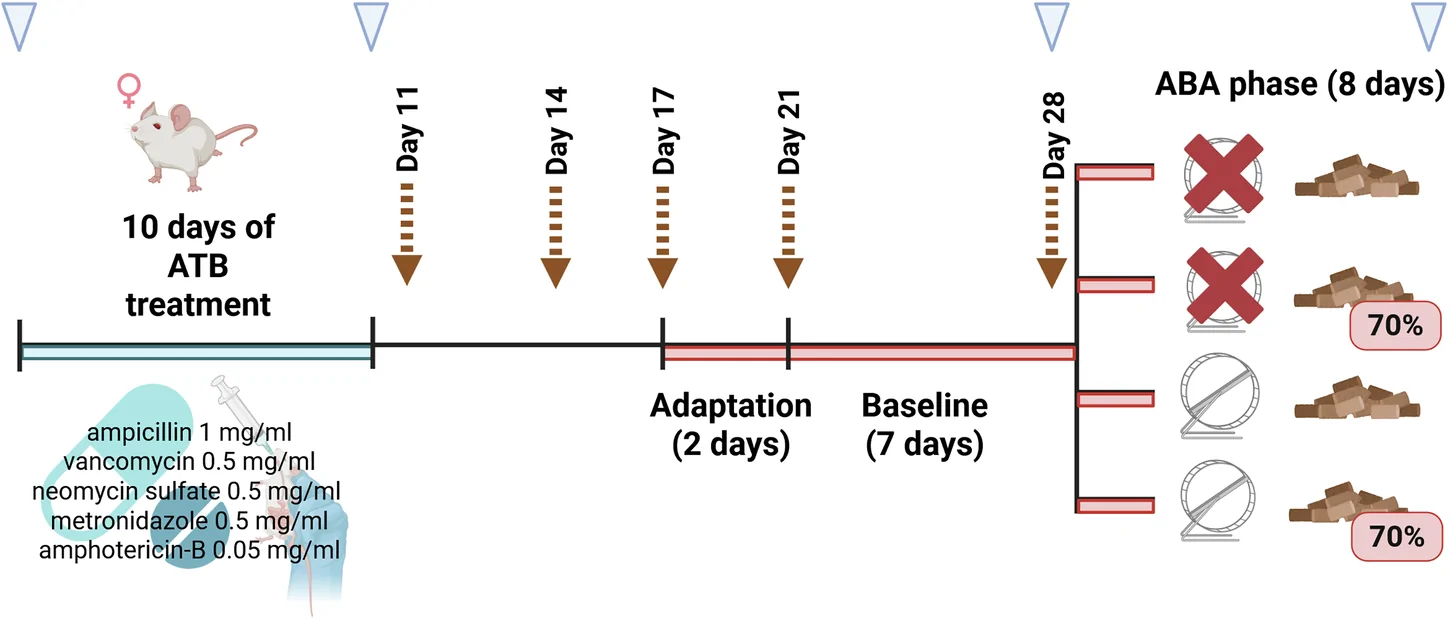
Design of the experiment. Mice were first treated with an antibiotic mixture. Then, five doses of FMT from healthy control, patients with acute AN, or patients with SEAN were administered (brown dashed arrows) before activity-based anorexia (ABA) induction. After the third dose, mice were separated into individual cages. Mice were adapted to single housing for two days. During the baseline phase, mice, their food intake, and running activity were monitored daily. During the ABA phase, some mice received 70% of their daily food portion, and some had access to a running wheel. Body weight and running activity were measured throughout the ABA phase. Fecal pellets were collected four times during the experiment, as indicated by blue arrows. Created in BioRender. Roubalova, R. (2026) https://BioRender.com/x61ugns.

Five grams of fresh stool in 50 ml of 0.9% saline solution were homogenized using an IKA ULTRA-TURRAX Tube Drive. The slurry was then passed through three cell strainers with pore sizes of 1000 µm, 500 µm, and 200 µm and centrifuged at 6000 × *g* for 15 min. The pellet was resuspended in 10 ml of 0.9% saline solution with 10% glycerol. Aliquots of 400 µl in cryogenic vials were immediately frozen at −80 °C and stored until use^13^. Preparation of the fecal microbiota for FMT was conducted under aerobic conditions. Therefore, we determined the differences in beta diversity (Bray–Curtis) between fresh-frozen stool and the fecal microbiota after sample processing using PERMANOVA with the *adonis2* function in R (Fig. S1). On the day of gut microbiota transplantation, aliquots from five individuals were thawed, pooled, and 200 µl of the pooled sample was immediately administered to mice by oral gavage according to the protocol design (Fig. 1).

### Design of the mouse experiment

This study was conducted in accordance with the ethical standards set by EU legislation on the use of experimental animals (2010/63/EU) and the Czech Animal Welfare Act. Animal protocols were approved by the Institute of Microbiology Animal Care and Use Committee (approval ID: 12/2023). All mice were obtained from Charles River Laboratories (Wilmington, MA, USA).

Mice were regularly fed at 4 pm with a maintenance diet for mice and rats (1324, Altromin). They were kept under controlled conditions with a 12- h light/12-hour dark cycle (lights on at 6 am) and a constant room temperature. The experiment was designed based on our previous study of the microbiome’s effect on ABA development^14^. Five-week-old female Balb/c mice were gavaged for ten days with 200 µl of an antibiotic mixture (ampicillin 1 mg/ml, vancomycin 0.5 mg/ml, neomycin sulfate 0.5 mg/ml, metronidazole 0.5 mg/ml, and amphotericin B 0.05 mg/ml) to decrease the microbial load in their guts. Five doses of 200 µl of pooled fecal microbiota from five healthy controls (ctrl), patients with acute AN (acute AN), or patients with chronic AN (SEAN) were administered by oral gavage (Fig. 1). After the third dose of fecal microbiota transplantation (FMT), mice were placed in individual cages to monitor food intake and activity. The mice were allowed to adapt to individual housing for two days. Half of the cages contained a wireless running wheel (wireless low-profile running wheel ENV-047, Med Associates, USA), which was blocked during the adaptation phase. During the baseline phase, the amount of food consumed, body weight, and running activity of each mouse were measured. During the activity-based anorexia (ABA) phase, half of the mice received 70% of their daily food portion. Running activity and body weight were again monitored during the ABA phase. The activity of mice was evaluated using Wheel Manager Data Acquisition software (Med Associates, USA).

In each experiment, twenty mice were included. The experiment was performed six times – twice for each type of microbiota (ctrl, acute AN, SEAN). Before the adaptation phase, mice were randomly divided into four groups of five to distinguish the effects of running wheel activity, food restriction, and the combination. These groups differed in the final (ABA) phase as follows: Group 1 – control mice without a running wheel and with food *ad libitum*; Group 2 – mice without a running wheel and with food restriction; Group 3 – mice with a running wheel and food *ad libitum*; and Group 4 – ABA (“anorexia-like”) mice with a running wheel and food restriction. Each experiment was repeated twice for each FMT group (ctrl, acute AN, SEAN).

During the experiment, stool samples were collected (1) at the beginning of the experiment before ATB treatment, (2) after 10 days of ATB treatment, (3) after five doses of FMT, and (4) at the end of the experiment to determine the microbiome and metabolome profiles in the gut of mice. At the end of the experiment, mice were euthanized by exsanguination, and their blood and brains were collected for subsequent experiments.

### Gut microbiome analysis

Fecal pellets from mice were collected throughout the experiment to determine shifts in gut microbiome after ATB administration, fecal microbiota transplantation, and ABA induction. Genomic DNA was isolated from murine fecal pellets using the ZymoBIOMICS DNA Miniprep Kit (Zymo Research, USA). Total genomic DNA was used to construct bacterial 16S rRNA amplicon sequencing libraries as previously described in Roubalova et al. (2024)^14^.

Briefly, libraries were constructed using a two-step PCR. In the first step, barcoding PCR, was performed with S-D-Bact-0341-b-S-17 (CCTACGGGNGGCWGCAG) and S-D-Bact-0785-a-A-21 (GACTACHVGGGTATCTAATCC) that amplified the V3–V4 variable region. The primers were extended at the 5´ ends with inline barcode sequences to increase multiplexing capacity. Additionally, overhang sequences were included in the primers to serve as priming sites for the second PCR step. Technical duplicates for each sample were prepared to account for PCR noise and sequencing stochasticity. To assess the performance of the metagenomics workflow, ZymoBIOMICS^TM^ microbial community standards and ZymoBIOMICS^TM^ microbial community DNA standards (Zymo Research, USA) were used. Products of the second PCR step were measured using QIAxcel Advanced capillary electrophoresis (Qiagen, Netherlands). The products were pooled equimolarly and purified with SpriSelect beads (Beckman Coulter, USA). The libraries were sequenced on a MiSeq platform (Illumina) and v3 chemistry (2 × 300 bp, 600-cycle kit) at GENEWIZ (Leipzig, Germany).

### Bioinformatic analysis

Raw paired-end reads were demultiplexed and trimmed using Skewer software^15^. The remainder of the pipeline was processed in RStudio (version 4.3.0). Trimmed reads were quality-filtered based on quality scores, with a maximum expected error per read of <2. High-quality reads were dereplicated, denoised, and chimeric sequences were removed using the DADA2 package^16^. Amplicon sequence variants (ASVs) were classified against the SILVA v138.2 reference database^17^ with a confidence threshold of 80. Based on a Procrustes analysis (*p* = 0.001, R = 0.967), we evaluated the similarities between technical duplicates and retained only ASVs consistently detected in both replicates. The bacterial dataset consisted of 17,976 ASVs represented by 11,382,338 high-quality reads, with a mean sequencing depth of 23,664 reads per sample (range: 2606–81,001).

### Effectiveness of ASV transfers from donors to recipients

To assess the transfer of donor-derived ASVs into recipient mice, we compared donor ASVs to raw sequencing reads from mouse fecal samples after FMT. Recipient reads were quality filtered using the same parameters as those applied to donor reads. Donor ASV sequences were exported as a FASTA file and compared with recipient reads using VSEARCH (v2.21.1) in the global alignment mode^18^. A match was defined as ≥99% sequence identity, with both forward and reverse strands considered. For each recipient, the recovery rate was calculated as the proportion of donor ASVs detected out of the total donor ASV pool. Group-level transfer efficiency was expressed as the mean recovery rate across mice within each FMT group (healthy controls, acute AN, SEAN). Transferred ASVs detected in recipients were then grouped by genus, and their relative abundances in the donor samples were summed to estimate each genus’s overall contribution. Genera were ranked by total abundance, and the top 20 most abundant donor genera successfully transferred are presented in Table S2.

### Statistical analysis of microbial composition

Prior to computing alpha diversity metrics, the ASV table was rarefied to the minimum sequencing depth. Alpha diversity was assessed using the Shannon index and ASV richness. These metrics were compared across sampling time points (start of the experiment, after ATB treatment, after FMT application, and end of the experiment) and experimental groups (mice transplanted with FMT from healthy controls, acute AN patients, or SEAN patients). Differences in Shannon diversity or observed ASVs among sampling or experimental groups were first assessed using the Kruskal–Wallis test. When significant, pairwise comparisons were performed using the Wilcoxon rank-sum test with Benjamini–Hochberg correction for multiple testing.

Beta diversity was visualized using principal coordinate analysis (PCoA). Differences in beta diversity among experimental groups were first assessed using PERMANOVA with the *adonis* function. To identify which specific group pairs contributed to the overall differences, pairwise PERMANOVA tests were performed using the *pairwise.adonis2* function. To assess the effects of sequencing batch, dietary restriction, activity, and their interactions, we fitted a multifactor PERMANOVA model using *adonis2* with 999 permutations, with terms added sequentially. A significant experimental batch effect was detected and was accounted for when interpreting the contributions of the other factors. Differential abundance analyses were conducted using LinDA software to assess changes in microbiota composition between the third sampling point (post-transplantation) and the fourth sampling point (post-treatment phase) of the experiment. The model included a three-way interaction, i.e., study time point × type of FMT (control, acute AN, SEAN) × experimental treatment (activity and/or dietary restrictions), which allowed a focus on within-individual microbiota changes and specifically tested whether the effects of the experimental treatment were moderated by the FMT donor group. Additional analyses, including only the two-way interaction between experimental treatment and study sample points, were performed to identify microbiota changes that occurred independently of transplant type. All LinDA models also included FMT donor identity as a covariate and mouse identity as a random effect. For data visualization and to facilitate interpretation of LinDA results, individual-level log_2_ fold changes between the third and fourth sampling points were calculated for each gut microbiota feature identified as significant. These log_2_ fold changes were then compared between treatment groups using Wilcoxon tests.

### LC-MS/MS analysis of short-chain fatty acids and neurotransmitter levels in mouse stool

To detect SCFA levels in stool samples, approximately 50 mg of mouse stool was mixed with 600 µL of 95% ethanol, 100 µL of Milli-Q water (Smart2Pure Water Purification System, Thermo Fisher Scientific, USA), and 175 µL of internal standard solution (150 µg/g of acetic-d4 acid and 15 µg/g each of propionic-d5, butyric-d7, isobutyric-d7, and isovaleric-d9) and homogenized. The homogenate was then centrifuged at 30,000 × g for 10 min at 4 °C.

For derivatization, 50 µL of supernatant was mixed with 50 µL of 50 mM 3-nitrophenylhydrazine hydrochloride, 50 µL of 50 mM N-(3-dimethylaminopropyl)-N′-ethyl carbodiimide hydrochloride, and 50 µL of pyridine (7% v/v). The mixture was incubated for 60 min at room temperature. Subsequently, 100 µL of formic acid (0.2% in 3:7 Milli-Q water:methanol) was added. Calibration samples were prepared from a mixture of SCFAs, and 20 µL of internal standard was added. Derivatization was performed using the same procedure as for stool samples. All chemicals used for derivatization and calibration were diluted in 3:7 Milli-Q water:methanol.

Targeted analysis was performed using an Agilent Infinity 1260 liquid chromatograph coupled to an Agilent 6470 LC/TQ mass spectrometer (Agilent Technologies, USA). Analytes were separated on a Kinetex Polar Phenyl-Hexyl 100 Å (2.6 μm; 100 × 2.1 mm) column (Phenomenex, USA) at 40 °C. The mobile phase consisted of phase A (0.1% formic acid in Milli-Q water) and phase B (0.1% formic acid in acetonitrile, LC-MS grade). Gradient elution was as follows (time [min]/% phase B): 0/15/0.35; 2/15/0.35; 5/25/0.35; 9/45/0.35; 9.10/100/0.40; 10.10/100/0.40; 10.50/15/0.35; 11.50/15/0.35. The injection volume was 1 μL. The matrix effect was minimized using automatic standard addition. Ionization was conducted by electrospray at 4500 V. The gas temperature was set to 230 °C, with a gas flow rate of 6 L/min. The nitrogen pressure on the nebulizer was 30 psi. The sheath gas temperature was 400 °C, and the flow rate was 12 L/min. Ion transitions and other mass spectrometric parameters were optimized using MassHunter Workstation Optimizer 10.0 and Source Optimizer 10.0 software (Agilent, USA). Additional analytical parameters are provided in Table S3.

To quantify neurotransmitter levels in stool, samples were processed as previously described^11^. Briefly, approximately 100 mg of stool was mixed with 1 mL of Milli-Q water, homogenized, centrifuged, and the supernatants were combined with 1 mL of LC-MS-grade acetonitrile (CHROMASOLV™, Honeywell, USA). Targeted analysis was performed using an Agilent Infinity 1260 liquid chromatograph coupled to an Agilent 6470 LC/TQ mass spectrometer. Analytes were separated on a Kinetex Polar C18 analytical column (2.6 μm, 3 mm × 100 mm) with a SecurityGuard Polar C18 precolumn (2.6 μm, 3 mm × 2 mm; Phenomenex, USA). Technical details, including calibration and validation metrics, are summarized in Table S4.

### RNA isolation, reverse transcription, and gene expression analysis of neuropeptides in hypothalami

At the end of the experiment, mouse brains were collected, hypothalami were excised, immersed in DNA/RNA Shield (Zymo Research, USA), and stored at −20 °C until use. RNA was isolated, reverse transcribed, and amplified as previously described^14^. Briefly, samples were homogenized using a FastPrep-24 instrument (MP Biomedicals, USA). Total RNA was extracted with TRIzol Reagent (Thermo Fisher Scientific, USA), treated with DNase (TURBO DNA-free Kit; Thermo Fisher Scientific, USA), and reverse transcribed with SuperScript IV Reverse Transcriptase (Thermo Fisher Scientific, USA). cDNA was amplified using a CFX96 Touch Real-Time PCR Detection System (Bio-Rad Laboratories, USA) and gb SG PCR Master Mix (Generi Biotech, Czech Republic). Gene expression data were normalized to two reference genes, hypoxanthine phosphoribosyltransferase (Hprt) and β-actin. Relative fold changes were calculated using the 2^−ΔΔCt method. In the hypothalamus, we quantified the expression of four orexigenic and two anorexigenic neuropeptides (the list of primers is provided in Table S5). Expression levels were related to the mean expression of samples from control mice fed *ad libitum* without access to a running wheel (Group 1) for each FMT donor group (ctrl, acute AN, SEAN).

### MALDI-MSI of neurotransmitters and metabolites

Matrix-assisted laser desorption/ionization mass spectrometry imaging (MALDI-MSI) was performed similarly to our previous study^19^. Brains were snap-frozen in pre-chilled isopentane in dry ice. Before cryosectioning, deeply frozen brain samples were allowed to equilibrate at −20 °C in a Leica CM1900 cryostat chamber (Wetzlar, Germany) for one hour. Striatal level sections (bregma +2.28 mm) of the mouse brain were cryosectioned according to the mouse stereotaxic atlas^20^. Consecutive 12-µm-thick brain sections for MALDI-MSI of neurotransmitters and metabolites^21^ were thaw-mounted onto precooled ITO and microscopic glass slides. The optimal brain section thickness was chosen based on Quast et al^22^. Slides were stored at −80 °C until analyzed. On the analysis day, sections were vacuum-desiccated for 30 min before and 15 min between and after internal-standards and matrix spraying. A mixture of deuterium-labeled standards (GABA-d6, 5-HT-d4, and DA-d4 at concentrations 10, 0.03, and 0.05 µg/ml) in 70% methanol was sprayed over tissue sections using a SunCollect sprayer (SunChrom, Germany) and an established protocol. Spraying parameters were: line distance, 2 mm; Z position, 35 mm; Z offset, 5 mm; layers, 10; flow rate, 15–30–45–60 μL/min; speed X/Y, Low10/High1. Samples were then subjected to on-tissue derivatization according to a previously published protocol^21^ and a modified spraying method. Briefly, FMP-10 solution (2.5 mg/ml) in 70% acetonitrile was sprayed over the tissue sections using a SunCollect sprayer and the following parameters: line distance, 2 mm; Z position, 25 mm; Z offset, 5 mm; layers, 26; flow rate, 10–20–45–60 μL/min; speed X/Y, Medium1/Medium1. Before MALDI-MSI analysis, the MALDI slide adapter with prepared glass slides was scanned using a flatbed optical scanner. MSI experiments were conducted using a SolariX 12T-2Ω MALDI FTICR MS instrument (Bruker Daltonics, MA, USA) equipped with a Smartbeam II 2 kHz laser. Data were acquired in positive-ion mode using quadrature phase detection (2 Ω) in a mass range of 150–1000 *m/z* and calibrated against red phosphorus clusters before analysis. The ion signal at *m/z* 555.2231 (FMP-10 ion cluster) was used for online calibration. The time-domain file size was set to 2 M, and ion optics were optimized for collision cell voltage (−1.5 V; DC bias, 0.7 V), time-of-flight delay (0.75 ms), and transfer optics (4 MHz; Q1, *m/z* 379). Laser power was tuned at the beginning of the experiment, and parameters were kept constant throughout data acquisition (200 laser shots per position). All MSI data were acquired at a 100 μm lateral resolution.

On-plate and on-tissue product-ion mass spectra of compounds of interest were acquired with compound-specific collision voltages (5-HT CV = 22 V, GABA CV = 28 V, DA CV = 22, Tyrosine CV = 19 V). Tyrosine standard was spotted on a MALDI target (1 µl) at a concentration of 10,000 ng/ml in 70% acetonitrile (ACN) premix with FMP10 (2.5 mg/ml in 70% ACN).

Images of metabolite distribution were generated using FlexImaging software (v.5.0; Bruker Daltonics, Germany), where regions of interest were annotated with reference to a stereotaxic atlas^20^. For relative quantitation, MSI datasets were processed in SCiLS Lab software (v.2019c Pro; Bruker Daltonics, Germany) and normalized to the corresponding deuterated internal standard. Ion mass spectra of 5-HT, GABA, and DA were verified against published literature^21^. For each analyte, the average area under the curve within the region of interest, averaged from both hemispheres, was used for data extrapolation.

## Results

### Gut microbiome of healthy controls and patients with anorexia nervosa

Our patients with acute AN and SEAN differed significantly in age and disease duration, which is consistent with disease progression. Most were taking medication, including antidepressants, antipsychotics, and anxiolytics (Table S1).

**Fig. 2:**
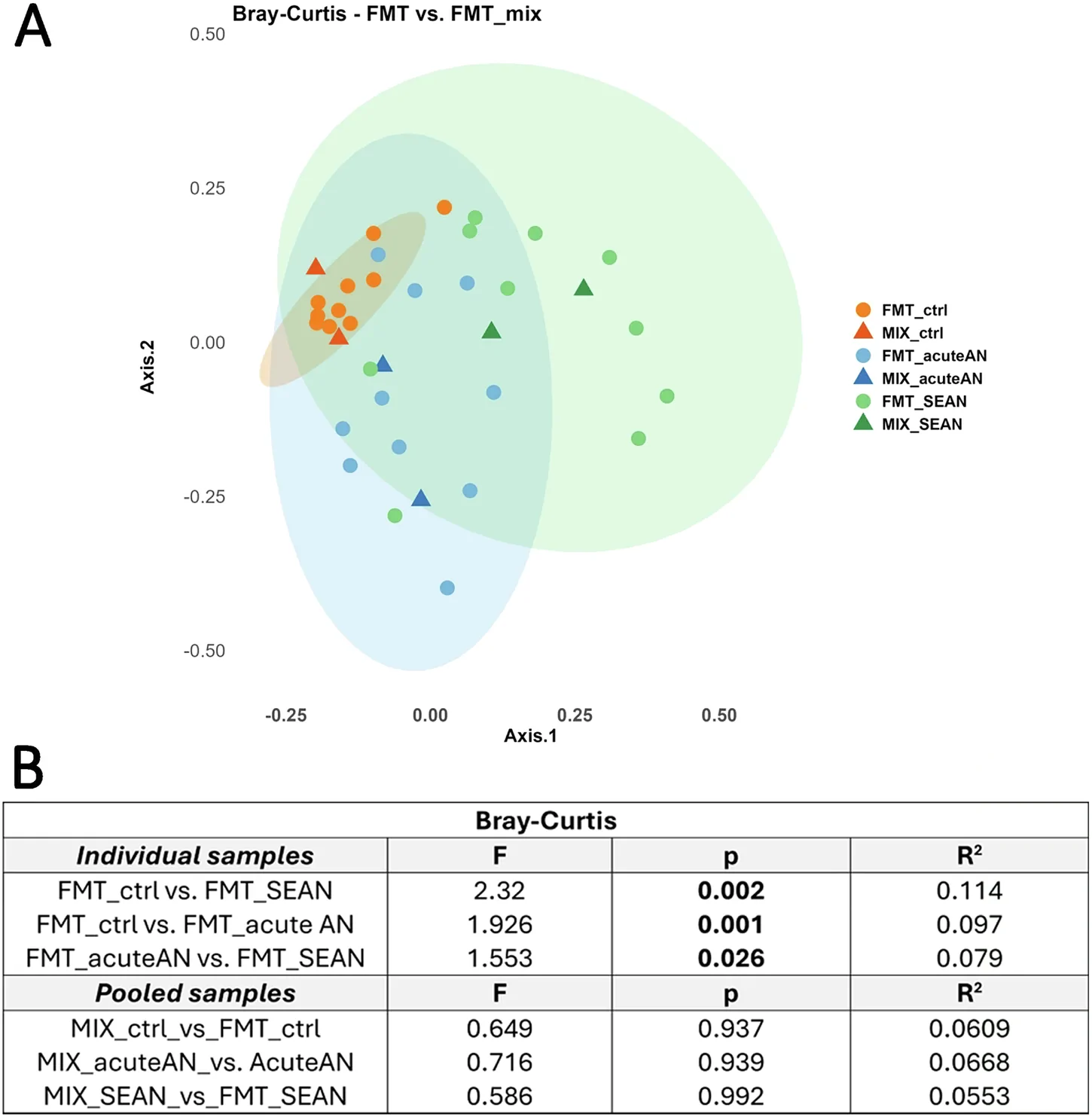
Variation in microbiota composition. **A** Principal coordinate analysis (PCoA) based on Bray–Curtis dissimilarities illustrates differences in microbiota composition among samples used for fecal microbiota transplantation. Circles (FMT) represent the microbiomes of individual human donor samples used for pooling (*n* = 10 in each donor group), whereas triangles (MIX) represent the microbiomes of pooled donor samples prepared for oral gavage (*n* = 2 in each donor group). **B** Statistical differences among groups were assessed using PERMANOVA with the adonis function in R.

We did not observe significant differences in gut microbiome alpha diversity among AN patient groups and healthy controls (data not shown). In contrast, microbial community composition differed significantly between healthy controls and patients based on Bray-Curtis dissimilarities, including clear distinctions between acute AN and SEAN patients (FMT_ctrl, FMT_acuteAN, FMT_SEAN). The beta diversity of pooled samples used for fecal microbiota transplantation (MIX_ctrl, MIX_acuteAN, MIX_SEAN) did not differ from the original individual samples within each group (Fig. 2).

### Change in the gut microbiome of mice during the experiment

**Fig. 3:**
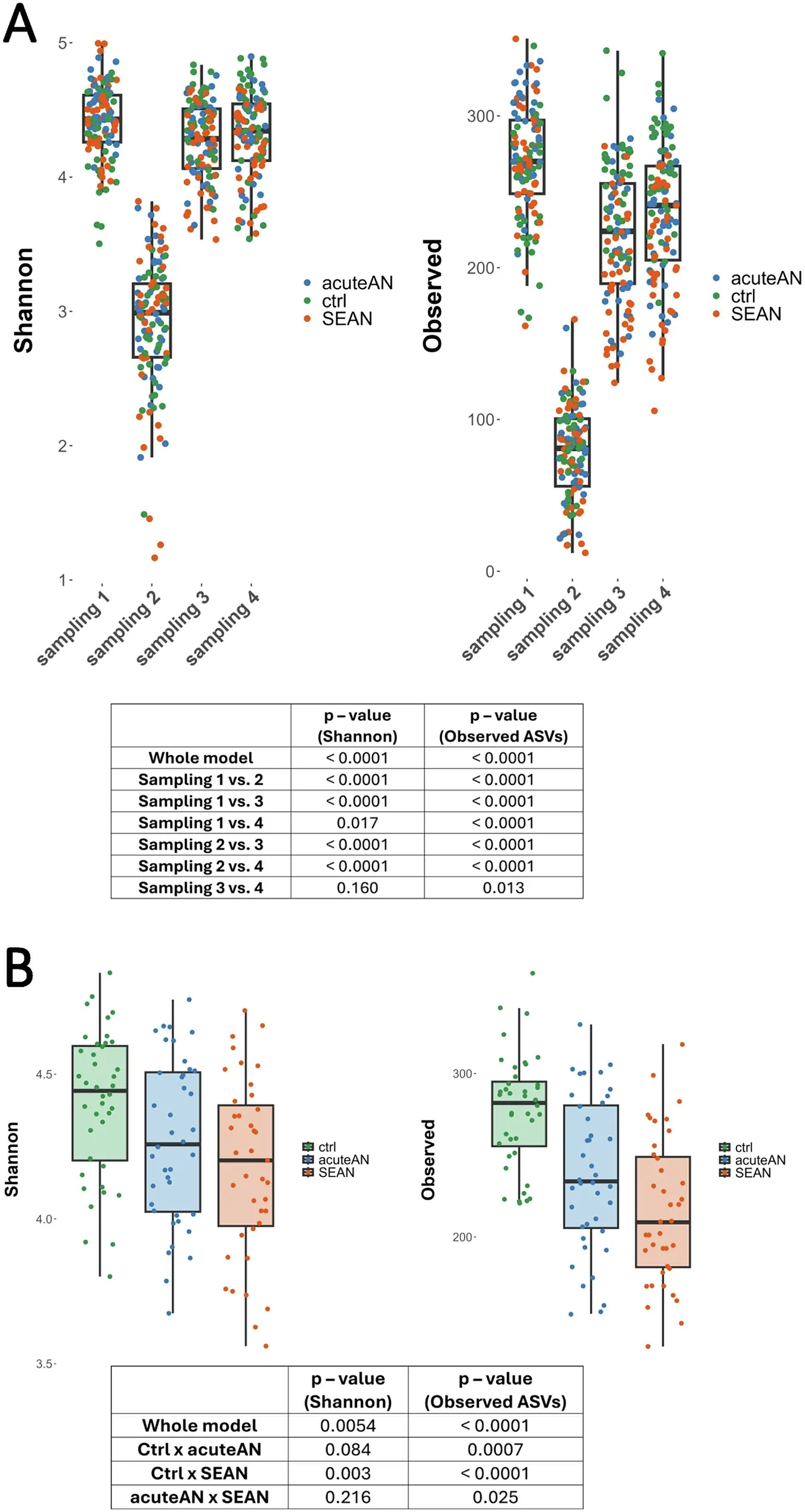
Changes in alpha diversity metrics during the experiment. **A** Differences in Shannon diversity and Observed ASVs across all sampling points. **B** Differences in Shannon diversity and Observed ASVs after FMT (sampling point 3) with microbiota from healthy controls (*n* = 40), acute AN (*n* = 40), or SEAN patients (*n* = 39). Differences were first assessed using the Kruskal–Wallis test. When significant, pairwise comparisons were conducted using the Wilcoxon rank-sum test with Benjamini–Hochberg correction for multiple testing. Sampling 1 – prior to ATB treatment; Sampling 2 – after ATB treatment; Sampling 3 – after five doses of FMT, Sampling 4 – at the end of the experiment. ctrl = healthy controls, acuteAN = acute anorexia nervosa, SEAN = severe and enduring anorexia nervosa.

Stool samples were collected from mice at four time points during the experiment: (1) at the beginning, before antibiotic treatment; (2) after antibiotic treatment; (3) after five doses of FMT; and (4) at the end of the experiment. As expected, the alpha diversity significantly decreased after 10 days of antibiotic treatment and increased again after microbiota transfer, as indicated by both the Shannon index and Observed ASVs (Fig. 3A). We also found significant differences in beta diversity among groups (Fig. S2A). After five doses of fecal microbiota from healthy controls, acute AN patients, or SEAN patients (sampling point 3), mice receiving microbiota from AN patients exhibited lower alpha diversity. This effect was most evident in Observed ASVs, where the mouse microbiomes differed significantly (Fig. 3B). Similarly, microbial community composition differed significantly among the three groups based on Bray-Curtis dissimilarities (Fig. S2B). The effectiveness of donor-derived microbiota transfer was assessed as both the percentage of transferred ASVs and the proportion of the donor community represented by these transferred ASVs in each FMT group (Table S6).

After five doses of FMT, mice were divided into four groups based on the food restriction and activity (i.e., access to a running wheel), as outlined in the experimental design (Fig. 1). At the end of the experiment, microbial community composition differed significantly among experimental groups, explaining 10% of the variance (F = 8.143, *p* = 0.001, R^2^ = 0.103). Both food restriction (F = 15.921, *p* = 0.001, R^2^ = 0.099) and activity (F = 3.368, *p* = 0.001, R^2^ = 0.021) independently and interactively (F = 2.892, *p* = 0.001, R^2^ = 0.018) influence the composition of the microbiota, with food restriction explaining about 10% of the variance and activity about 2%. Interestingly, interaction indicates that the combination of food restriction and activity affected the microbiome in a non-additive way (F = 2.129, *p* = 0.002, R^2^ = 0.0265). A sequencing batch effect was also detected (F = 7.670, *p* = 0.001, R^2^ = 0.048), which explains about 5% of the total variance.

**Fig. 4:**
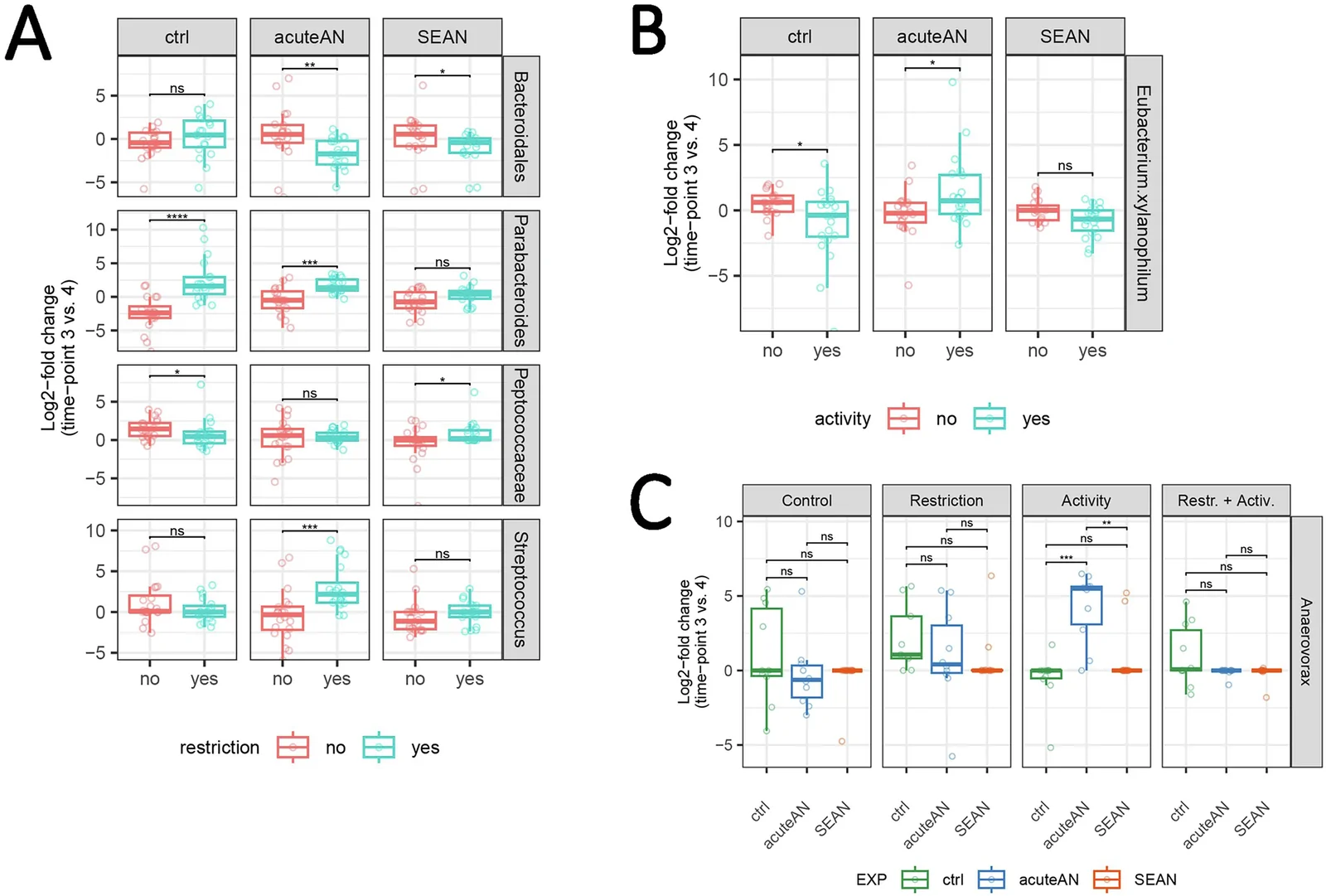
Changes in microbial taxa reflecting exposure to food restriction, activity, or their combination. Longitudinal within-individual log_2_ fold changes are shown for microbial taxa whose temporal variation was significantly affected by: **A** the combined effect of food restriction and FMT type (significant time point × transplant type × food restriction interaction in LinDA); **B** the combined effect of activity and FMT type of (significant time point × transplant type × activity interaction in LinDA); **C** the combined effect of food restriction and activity together with FMT type (significant time point × transplant type × combined treatment interaction in LinDA). Group differences in log_2_ fold change were assessed using Wilcoxon tests.

Furthermore, we determined the differential microbial abundance related to food restriction, activity, and their interaction. The analyses identified four taxonomic features (Bacteroidales, *Parabacteroides*, Peptococcaceae, and *Streptococcus*) whose temporal changes during the ABA induction phase (difference between sampling points 3 and 4) were influenced by the combined effect of food restriction and FMT type (i.e., the study time point × FMT type × food restriction three-way interaction; Fig. 4A). In contrast, parallel analyses revealed only a single taxonomic feature, *Eubacterium xylanophilum*, that responded differently to the activity treatment depending on the FMT source (Fig. 4B). When the combined effect of activity and food restriction was incorporated into the model, only the genus *Anaerovorax* showed an FMT-specific increase in abundance in mice fed *ad libitum* and provided with a running wheel, transplanted with microbiota from acute AN patients (Fig. 4C).

Analyses testing only the two-way interactions (i.e., excluding FMT type) showed that food restriction had a stronger effect on gut microbiota composition than activity. Food restriction was associated with significant temporal changes in 19 microbial taxa (Fig. S3), whereas activity affected only a single taxon (Fig. S4).

### Levels of short-chain fatty acids in the stool of mice during the experiment

**Fig. 5:**
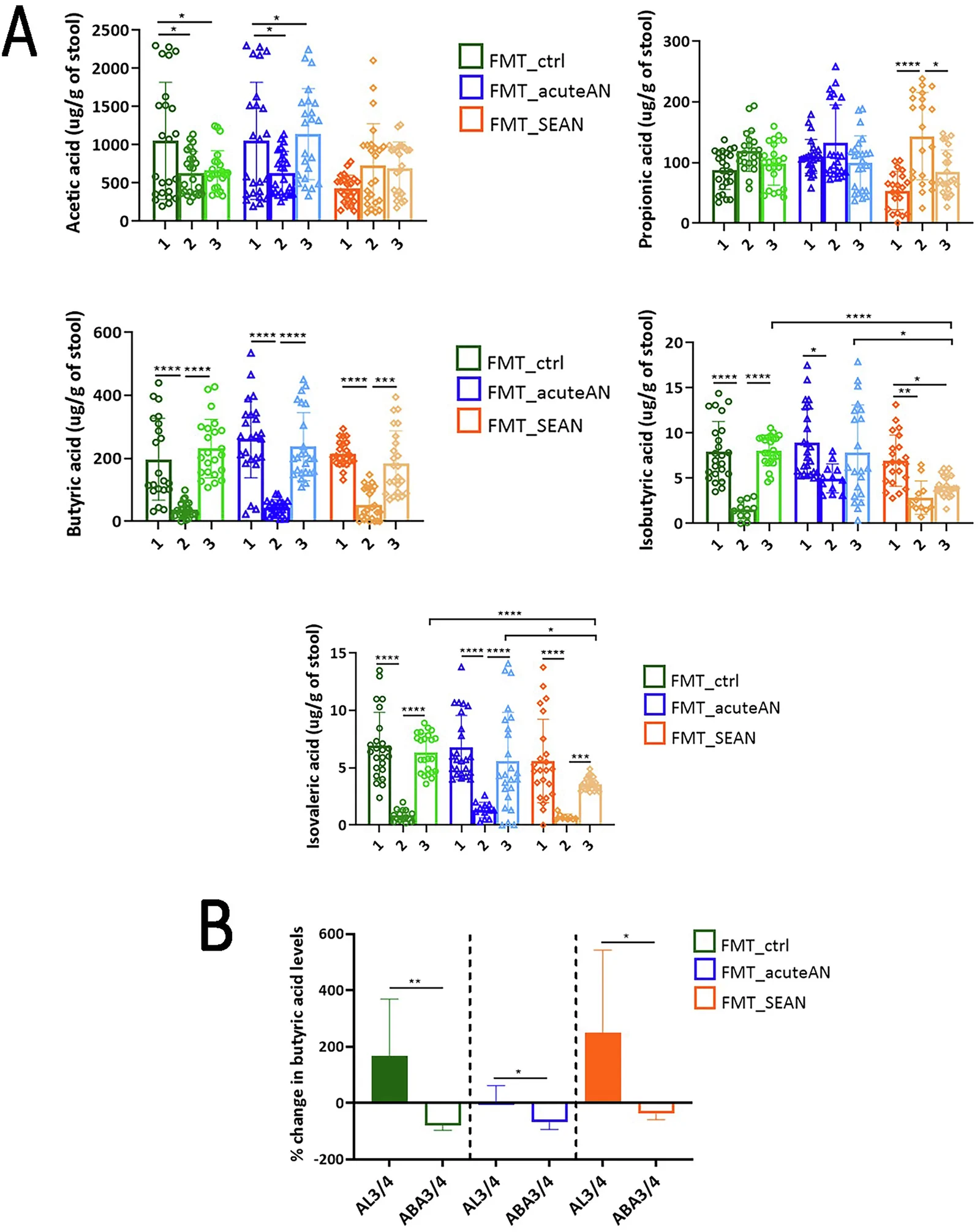
Fecal SCFA levels during the experiment. **A** Levels of acetic, propionic, butyric, isobutyric, and isovaleric acids at the beginning of the experiment (1), after ATB treatment (2), and after five doses of FMT (3); *n* = 24. **B** Changes in butyric acid levels during the ABA phase (sampling points 3 and 4) in mice fed *ad libitum* (AL) and in ABA mice (food restriction, running wheel access). The differences were assessed using the Kruskal-Wallis test with Dunn´s multiple comparisons test (**A**) or Mann-Whitney test (**B**). *p < 0.05, **p < 0.01.***p < 0.001, ****p < 0.0001.

SCFA levels were assessed in stool samples collected at the same time points as the microbiome. We determined the levels of five SCFAs (acetic, butyric, propionic, isobutyric, and isovaleric acids). A significant decrease in SCFA levels was observed after ATB treatment, except for acetic acid in mice transplanted with SEAN microbiota and propionic acid across all groups (Fig. 5A). Following five doses of fecal microbiota (sampling point 3), SCFA levels increased in all mice. However, we did not detect substantial differences in acetic, propionic, or butyric acid levels among mice receiving microbiota from healthy controls, acute AN, or SEAN patients. In contrast, mice transplanted with FMT from SEAN patients had significantly lower levels of isobutyric and isovaleric acids (Fig. 5A).

In the final phase of the experiment, mice were divided into four groups based on their access to food and a running wheel (Fig. 1). Between sampling points 3 and 4, we observed a significant decrease in butyric acid levels in ABA mice (Fig. 5B). This decrease was not caused by food restriction or activity alone, but by the combined effect (Fig. S5A). The remaining detected SCFAs (acetic, propionic, isobutyric, and isovaleric acids) did not show significant changes during ABA induction (Fig. S5B).

### Levels of GABA, serotonin, and tryptophan in the stool of mice during the experiment

**Fig. 6:**
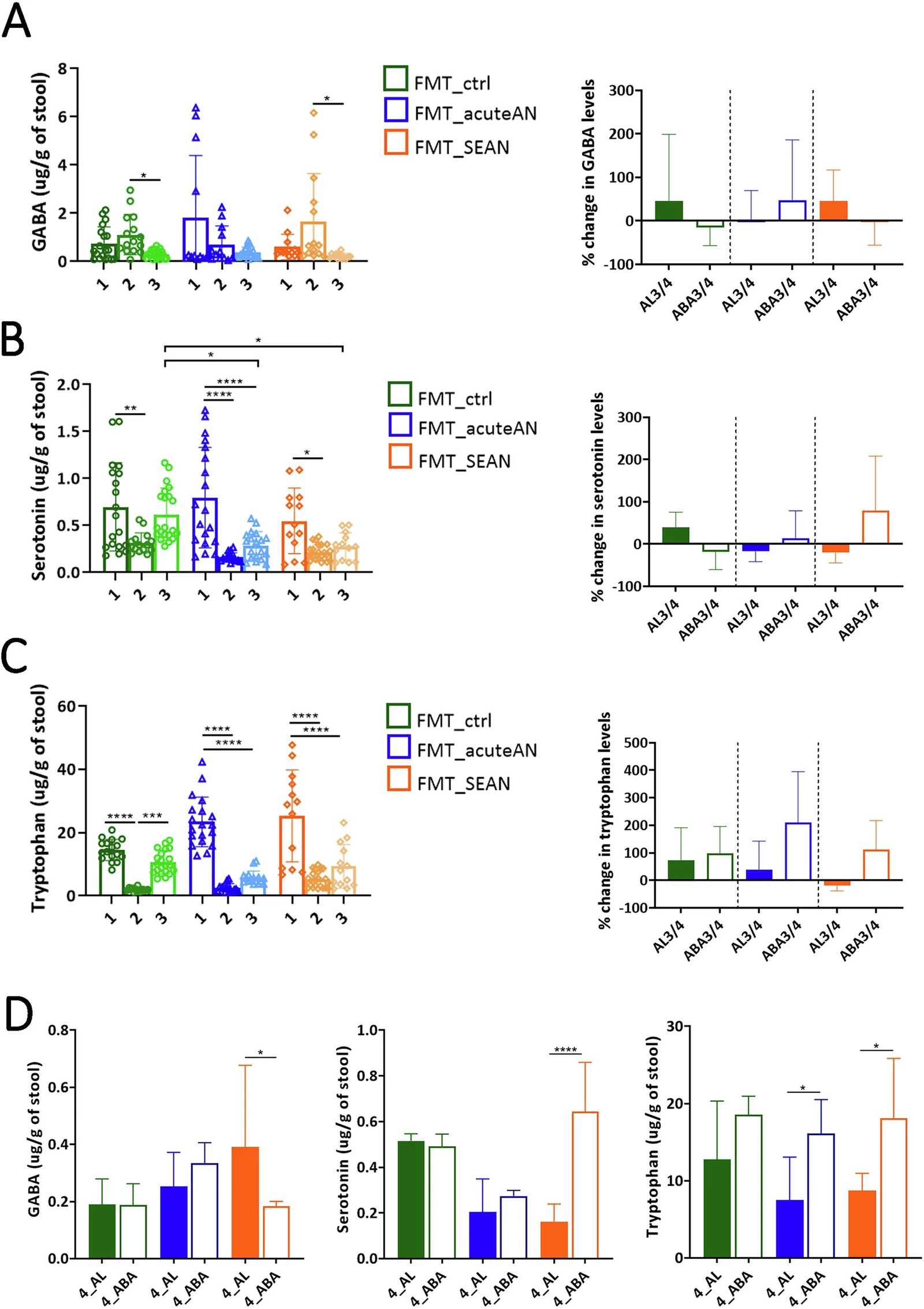
Dynamics of fecal neuroactive metabolites during the experiment. The fecal levels of GABA (**A**), serotonin (**B**), and tryptophan (**C**) during the experiment, and at the end of the experiment (**D**). Neurotransmitter levels were measured (1) before ATB treatment, (2) after ATB treatment, (3) after five doses of FMT, and (4) after ABA induction. Differences in the levels of neurotransmitters were assessed using the Kruskal-Wallis test with Dunn´s multiple comparison test. Percent changes in neurotransmitter levels were assessed using the Mann-Whitney test. *p < 0.05, **p < 0.01.***p < 0.001, ****p < 0.0001. AL = mice fed *ad libitum*; ABA = activity-based anorexia (food restriction, running wheel access).

We measured the levels of GABA, serotonin, and its precursor tryptophan in fecal samples collected at the same sampling points as the microbiome and SCFAs. GABA levels did not change significantly after ATB-treatment (sampling point 2), but we observed a significant decrease after transplantation of microbiota from healthy controls and SEAN patients (sampling point 3). During the ABA induction phase, no substantial changes in GABA levels were detected (Fig. 6A).

Serotonin and tryptophan levels decreased significantly after ATB treatment. Following FMT, these levels were restored in mice that received microbiota from healthy controls. In contrast, mice transplanted with microbiota from AN patients (both acute and SEAN) did not recover their original fecal serotonin or tryptophan levels (Fig. 6B, C). During the ABA phase, serotonin and tryptophan levels increased in ABA mice transplanted with patient-derived microbiota; however, due to high variability in neurotransmitter levels, these changes were not statistically significant. Additionally, ABA mice receiving microbiota from SEAN patients exhibited higher serotonin and tryptophan levels and lower GABA levels compared with *ad libitum*–fed mice. Tryptophan levels were also significantly higher in ABA mice transplanted with microbiota from acute AN patients compared with *ad libitum*–fed mice (Fig. 6D).

### The difference in behavioral patterns in mice receiving FMT from healthy controls or from patients with acute or chronic AN

**Fig. 7:**
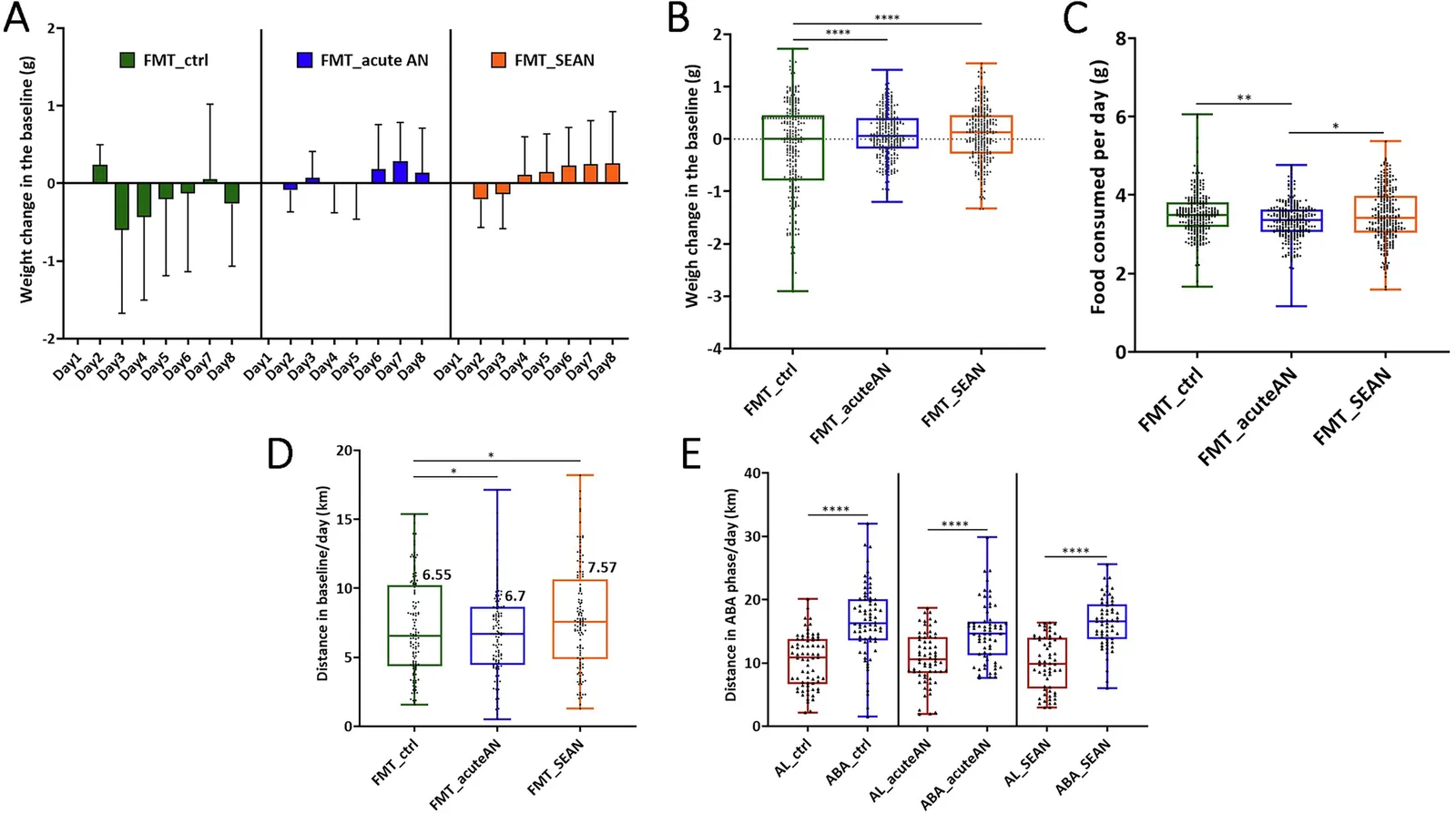
Differences in body weight and activity of mice after FMT from healthy controls or from patients with acute or chronic (SEAN) AN. **A** Daily weight change of mice after FMT during the baseline phase. **B** Cumulative weight change during the baseline phase. **C** Daily food intake during the baseline phase. **D** Distance run by mice during the baseline phase (values represent median kilometers run per day). **E** Running activity of food *ad libitum* (AL) and food-restricted (ABA) mice during the ABA phase. Differences among groups were assessed using one-way ANOVA with Tukey´s multiple-comparisons test. *p < 0.05, **p < 0.01, ****p < 0.0001. FMT = fecal microbiota transplantation.

After five doses of FMT, we monitored body weight, food intake, and running activity of individual mice during the baseline phase of the experiment, when all mice were provided food *ad libitum* (Fig. 7). Weight gain was significantly higher in mice transplanted with microbiota from patients with the acute and chronic forms of AN than in mice receiving FMT from healthy controls (Fig. 7A, B). However, mice transplanted with microbiota from acute AN patients consumed significantly less food than mice transplanted with control microbiota (adjusted *p* = 0.0064) and those transplanted with microbiota from SEAN patients (adjusted *p* = 0.0337; Fig. 7 C). Running activity during the baseline phase was highest in mice transplanted with microbiota from SEAN patients (Fig. 7D). Activity during the ABA phase, when half of the mice in each FMT group received only 70% of their daily food portion, was significantly higher in the food-restricted mice than in the mice with food *ad libitum*, demonstrating successful induction of the activity-based anorexia model. However, we did not observe any significant effect of the FMT source on running activity during this phase of the experiment (Fig. 7E). Consistent with this, the negative association between body weight and daily running distance was highly significant across all FMT groups (Fig. S6), with the strongest Spearman correlation observed in mice transplanted with microbiota from healthy controls.

**Fig. 8:**
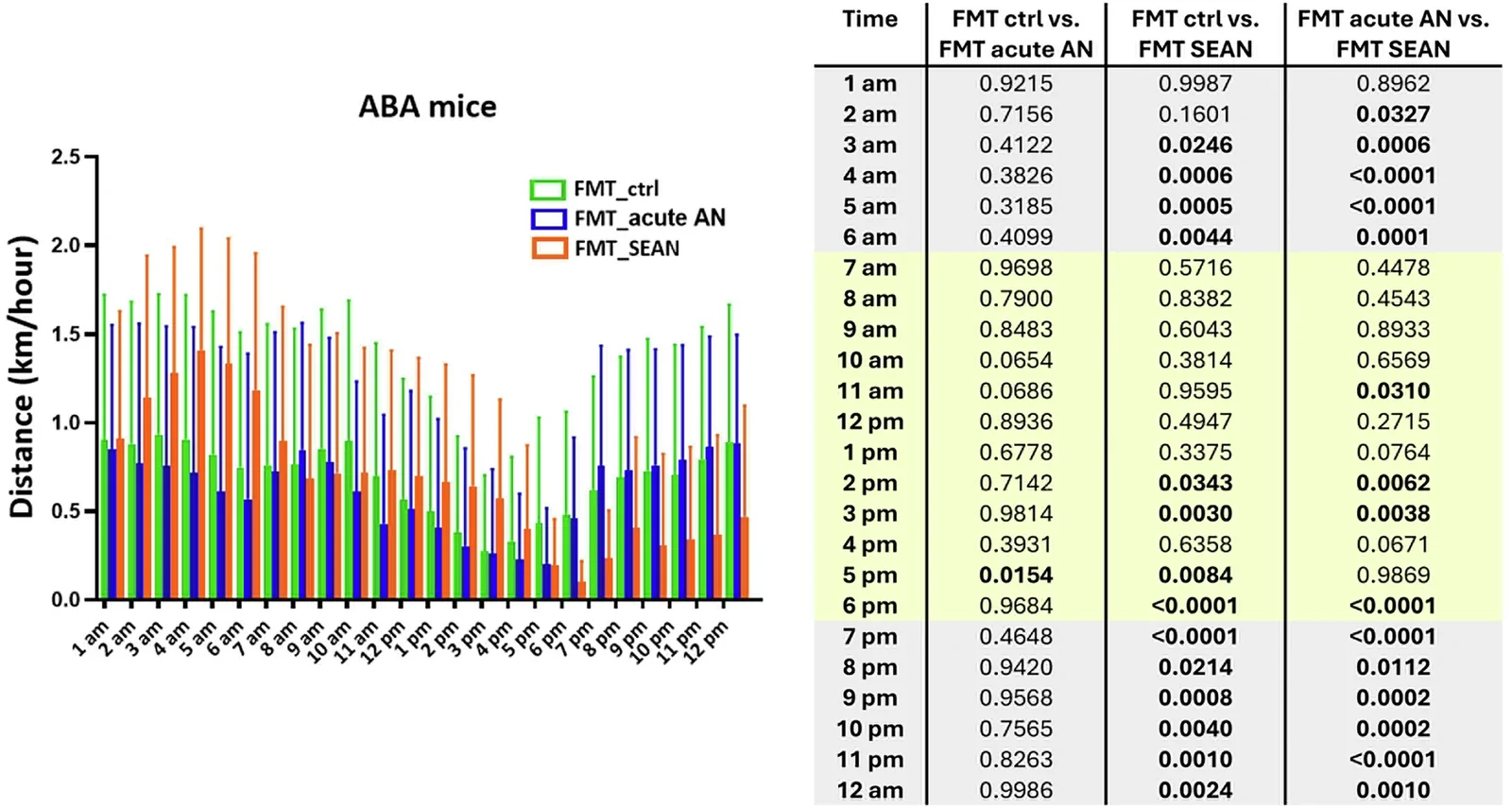
Activity of ABA mice receiving different transplanted microbiota during the ABA phase. Data were analyzed using a mixed-effects model followed by Tukey’s multiple-comparisons test. ctrl = FMT from healthy controls; acute AN = FMT from patients with acute anorexia nervosa; SEAN = FMT from patients with severe and enduring anorexia nervosa.

During the ABA phase, diurnal running-wheel activity differed among mice receiving microbiota from healthy controls or AN patients (Fig. 8, Fig. S7). Significant differences were observed between the FMT SEAN group, which showed the highest activity between 3 am and 6 am and the lowest activity between 5 pm and 10 pm, and the FMT ctrl and FMT acute AN groups. Running-wheel activity did not differ significantly between the latter two groups.

### The effect of FMT on appetite regulation

**Fig. 9:**
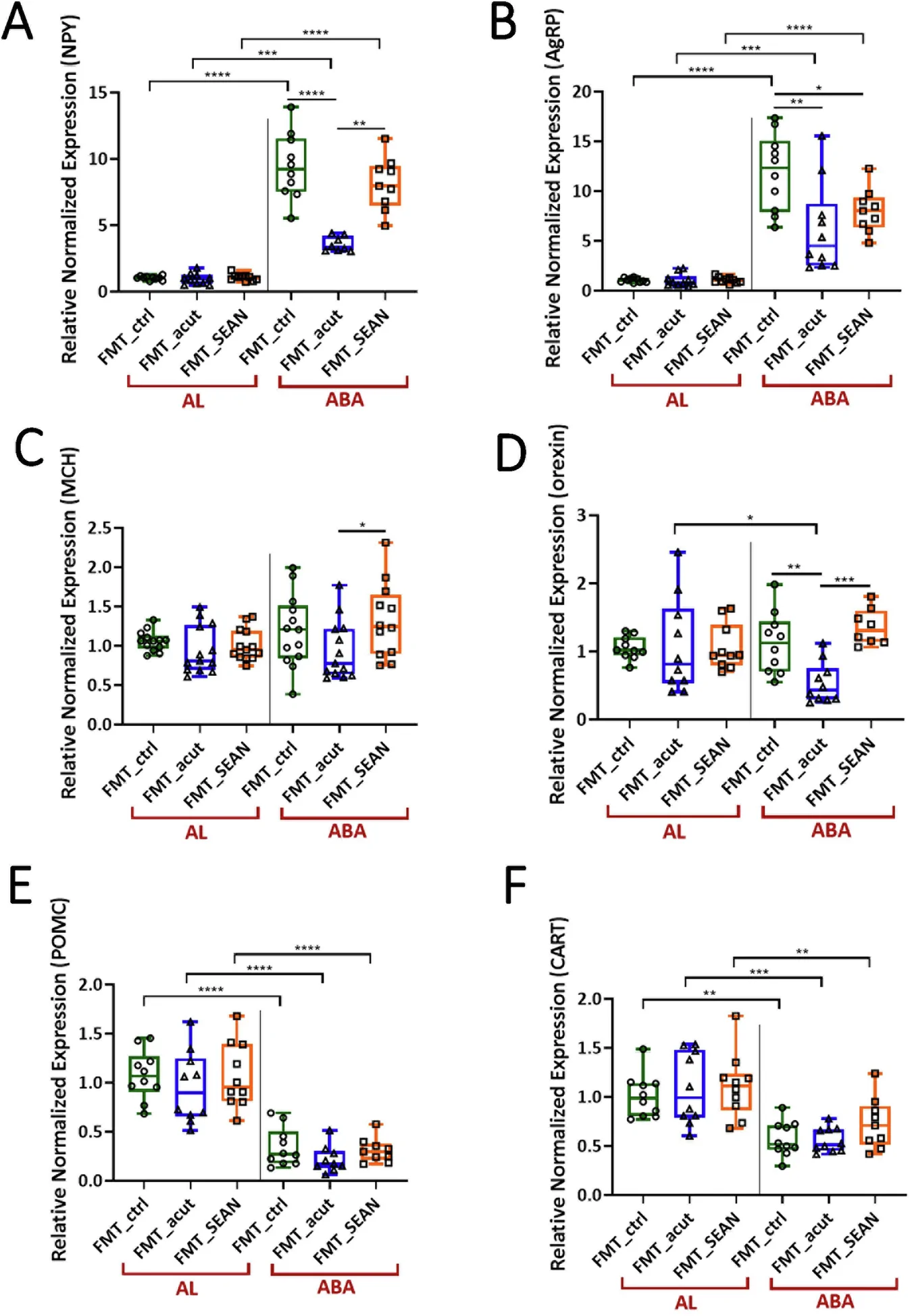
Expression of orexigenic and anorexigenic peptides in mice with different microbiota. Levels of: **A** neuropeptide Y (NPY), **B** agouti-related peptide (AgRP), **C** melanin-concentrating hormone (MCH), **D** orexin, **E** pro-opiomelanocortin (POMC), and **F** corticotropin-releasing hormone (CRH) in the control mice fed *ad libitum* without running-wheel access (AL) and “AN-like” mice with running-wheel and food restriction (ABA). Differences among groups were assessed using one-way ANOVA with Sidak´s multiple-comparisons test. *p < 0.05, **p < 0.01, ***p < 0.001, ****p < 0.0001.

The impact of microbiota on hypothalamic appetite regulation was assessed at the end of each experiment. We measured the levels of four orexigenic peptides (NPY, AgRP, MCH, and orexin) and two anorexigenic peptides (POMC and CART) expressed in the hypothalamus. For the two most potent orexigenic molecules, NPY and AgRP, we observed significant effects of both food availability (food *ad libitum* or food restriction; Fig. S8) and microbiota source (Fig. 9). These effects were less pronounced than for the other peptides. We observed decreased levels of orexin in ABA mice with microbiota transplanted from acute AN patients (Fig. 9C, D). However, this reduction was more likely attributable to increased physical activity than to ABA induction, as a similar pattern was observed in *ad libitum*–fed mice with running-wheel access (Fig. S8). Levels of the two potent anorexigenic molecules, POMC and CART, were significantly reduced in food-restricted mice, with no detectable microbiota-specific effects (Fig. 9E, F).

### Changes in brain metabolites during ABA induction

To further investigate neurotransmitter involvement in ABA development, we used MALDI-MSI (Matrix-Assisted Laser Desorption/Ionization-Mass Spectrometry Imaging) to visualize their spatial distribution in brain sections. We focused on the caudate putamen, a striatal region implicated in feeding regulation. We quantified serotonin, GABA, dopamine, its precursor tyrosine, and several downstream metabolites, DOPAL (3,4-dihydroxyphenylacetaldehyde), DOPAC (3,4-dihydroxyphenylacetic acid), 3-MT (3-methoxytyramine), and homovanillic acid.

**Fig. 10:**
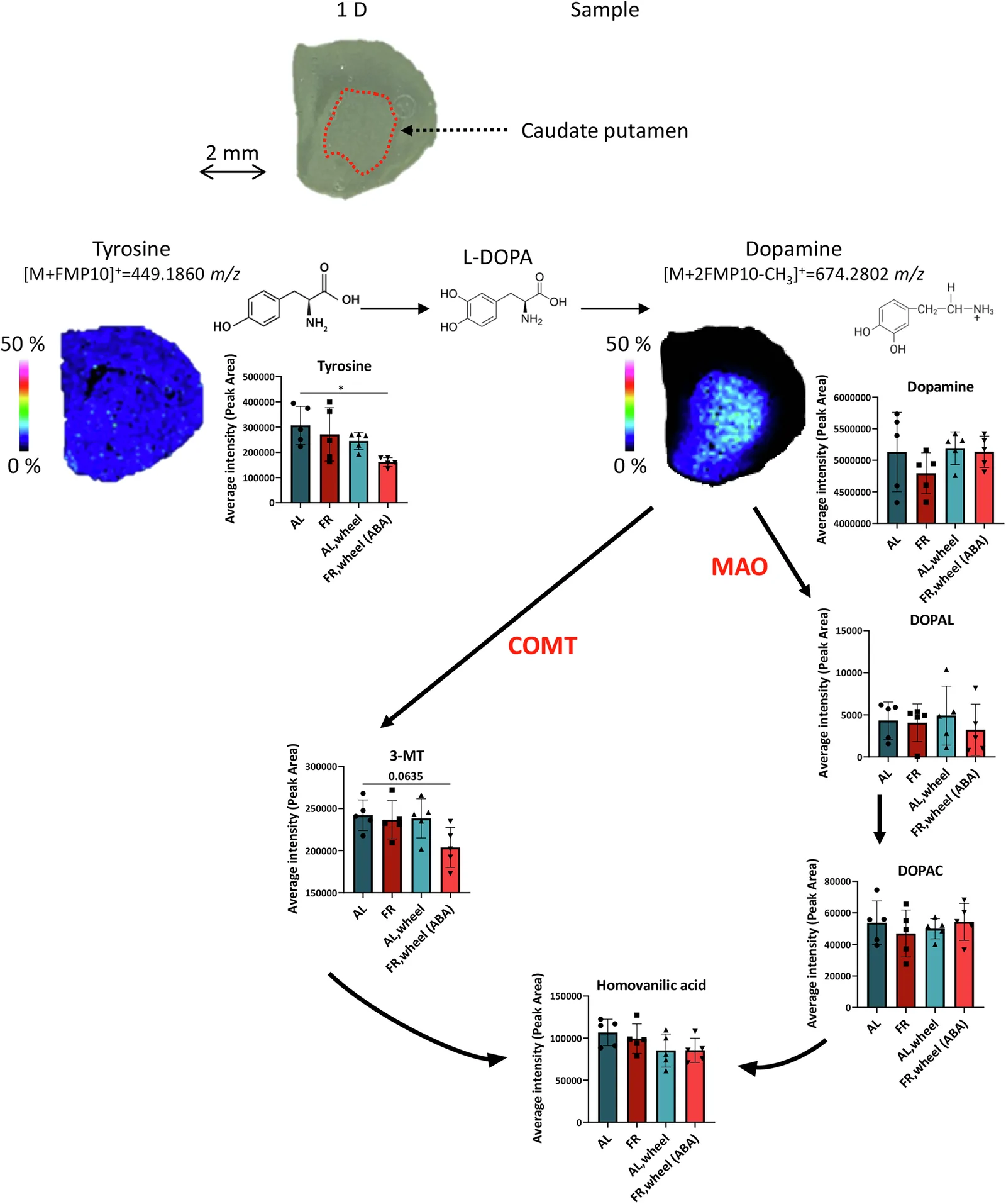
Changes in dopamine-related metabolic pathways in the caudate putamen of activity-based anorexia mice (*n* = 5). The brain section illustrates the anatomical regions in which metabolites were quantified. MALDI-MSI images show tyrosine and dopamine levels normalized to their corresponding deuterated internal standards. Data were analyzed using one-way ANOVA followed by Tukey’s multiple-comparison test. Asterisks indicate statistical significance: *p < 0.05. Graphs represent means with standard deviations. AL = *ad libitum*, FR = food restriction, COMT = catechol-*O*-methyltransferase, MAO = monoamine oxidases, 3-MT = 3-methoxytyramine, DOPAL = 3,4-dihydroxyphenylacetaldehyde, DOPAC = 3,4-dihydroxyphenylacetic acid.

Tyrosine levels were significantly reduced in “AN-like” mice subjected to food restriction and running-wheel access. The dopamine metabolite 3-MT also showed a marked, though not statistically significant, decrease in these mice (Fig. 10). No significant differences were detected in serotonin or GABA levels. Notably, mass spectrometry imaging was performed only on brain sections from conventional mice without FMT that underwent ABA induction.

## Discussion

The composition of the gut microbiome is consistently reported to be altered in patients with anorexia nervosa. Additionally, the levels of microbial metabolites that may affect gut–brain axis signaling (such as SCFAs and neurotransmitters) were also found to be altered in AN patients^10^. Most studies compared healthy controls with patients with anorexia nervosa (AN). Recently, we showed that the microbiota composition and fecal microbial metabolic profile differ not only between healthy controls and AN patients, but also between patients with acute AN and those with chronic severe and enduring AN (SEAN)^11^. In the present study, we followed up on this finding to clarify the contribution of gut microbiota to disease development and progression. First, we screened the gut microbiome of healthy controls and acute and chronic AN patients whose samples were used for fecal microbiota transfer (FMT) into mice. In accordance with our previous studies, we found greater interindividual variability in patients with AN than in healthy controls^11,23^. Distinct beta diversity was observed not only between healthy controls and patients, but also between acute AN and SEAN patient groups. Additionally, the pooled gut microbiota used for FMT (healthy controls (HC), patients with acute AN, or SEAN) clustered with the individual samples from the corresponding groups (Fig. 2). After five FMTs into mice, we found significantly lower alpha diversity of the gut microbiome in mice transplanted with microbiota from AN patients compared to those transplanted with microbiota from HC. In addition, ASV richness was significantly lower in mice transplanted with microbiota from SEAN patients than in those transplanted with microbiota from acute AN patients (Fig. 3B). Microbial community composition also differed significantly among the three groups of mice based on Bray-Curtis dissimilarities (Fig. S2B). These results are consistent with our previous data from a larger cohort of HC and AN patients^11^.

After inducing activity-based anorexia in mice with distinct gut microbiomes, we found that food restriction, activity, or their combination influenced the composition of the gut microbiota (Fig. 4). We also determined that the combined effect of food restriction and FMT type influenced the abundance of four bacterial taxa: the order Bacteroidales, the family Peptococcaceae, and the genera *Parabacteroides* and *Streptococcus* (Fig. 4A). The order Bacteroidales is among the most abundant colonizers of the human gut. It includes several ubiquitous and stable bacterial species that encode many carbohydrate-active enzymes, enabling them to utilize dietary polysaccharides and produce short-chain fatty acids^24^. These molecules are important for maintaining the intestinal barrier and gut immune homeostasis. Importantly for the development and progression of eating disorders, SCFAs may interact with free fatty acid receptors (FFARs) on the surface of enteroendocrine cells and influence the production of peripheral appetite regulators such as peptide YY (PYY) and glucagon-like peptide-1 (GLP-1). SCFAs that enter the bloodstream may also cross the blood-brain barrier and affect central appetite regulation in the arcuate nucleus of the hypothalamus^25^. Food restriction specifically reduced the relative abundance of the order Bacteroidales in mice colonized with microbiota from both acute and chronic AN patients. This decrease may be associated not only with the reduced appetite observed in ABA mice transplanted with acute AN microbiota (Fig. 7), but also with the increased gut permeability and proinflammatory response previously reported in ABA mice in several studies^26,27^. The abundance of *Parabacteroides*, a member of the order Bacteroidales, increased significantly following food restriction in mice colonized with microbiota from healthy controls and acute AN patients, but remained unchanged in mice colonized with microbiota from SEAN patients. *Parabacteroides* is a core member of the human gut microbiome, affecting intestinal physiological processes such as energy intake, immune system homeostasis, and hormone secretion. Generally, metabolites from this genus are associated with alleviating the progression of certain diseases, such as rheumatoid arthritis, chronic obstructive pulmonary disease, and metabolic dysfunction-associated steatotic liver disease^28^. In addition, we found significantly increased levels of the genus *Streptococcus* in food-restricted mice transplanted with microbiota from acute AN patients compared to mice fed *ad libitum*. Certain *Streptococcus* species are reported to produce serotonin, a key regulator of gastrointestinal motility and secretion. Some reports also suggest that serotonin may act as a pro-inflammatory molecule in the gut^29^. Gastrointestinal symptoms are highly prevalent in AN, and in our previous study, we reported dysregulated serotonin levels in patients with AN compared to healthy controls; however, in that study, we did not distinguish between acute and chronic AN patients^23^. Importantly, microbiota-derived serotonin represents only a very small fraction of the intestinal serotonin pool; therefore, its physiological effect on the gastrointestinal tract remains uncertain. In contrast, significantly altered levels of the family *Peptostreptococcaceae* were found in food-restricted mice with microbiota from HC and SEAN patients, but not in those colonized with microbiota from acute AN patients. Specifically, this family was decreased in food-restricted mice compared to *ad libitum*-fed mice colonized with microbiota from healthy controls, whereas the opposite was observed in mice colonized with microbiota from SEAN patients. A Mendelian randomization analysis assessing the causal effect of gut microbiota on AN risk identified the family *Peptostreptococcaceae* as one of the bacterial taxa potentially increasing the risk of AN development^30^. However, our results do not support these findings in the mouse model of AN since we observed no significant differences in the development of the ABA phenotype across the different FMT groups.

The interaction analysis assessing differential microbial abundance as a function of activity across distinct microbial colonization identified a single species, *Eubacterium xylanophilum* (Fig. 4B). The genus *Eubacterium* includes SCFA-producing species, and some studies have reported an increased abundance of this genus in association with higher physical activity^31,32^. Nevertheless, in our study, differences in *Eubacterium xylanophilum* abundance in mice with access to a running wheel were specific to individual FMT groups, making it difficult to draw direct conclusions about the relevance of these changes. When we introduced the combined effect of food restriction and activity in our model, we found a significant increase in the abundance of the genus *Anaerovorax* in mice colonized with microbiota from acute AN patients and given access to a running wheel (Fig. 4C).

Generally, changes in microbial abundance were primarily driven by food restriction rather than by activity alone or by the combination of the two. However, having only ten mice per FMT group in the ABA phase (based on food intake and presence of a running wheel) was not sufficient to detect some subtle yet important variations in gut microbiota composition. The alterations in gut microbiomes observed in our FMT mouse groups may be linked to dysregulated appetite, impaired intestinal permeability, and a dysregulated immune system reported in patients with AN^11,33–35^. Thus, the microbiome may underlie the complex interplay between disease progression and gastrointestinal health.

SCFAs have consistently been reported as decreased in the feces of patients with AN^11,23,36^, most likely due to insufficient intake of polysaccharides available for microbial fermentation. Low levels of SCFAs may correspond to poor intestinal health and chronic pro-inflammatory tuning of the immune system, not only in patients with AN, but also in other psychiatric diagnoses such as depression and anxiety, which are common comorbidities of AN^37,38^.

To our knowledge, only one publication has examined SCFA levels in mice in the context of a mouse model of anorexia nervosa. This study recently reported altered SCFA levels in germ-free mice transplanted with microbiota derived from individuals with AN or healthy controls. Acetate significantly decreased and butyrate increased in mice colonized with AN-microbiota compared to those with control microbiota^39^. In our study, we observed a clear drop in all measured SCFA levels after the antibiotic treatment, with the exception of propionic acid (Fig. 5A). Following FMT, SCFA levels were restored; however, isobutyric and isovaleric acid levels were significantly lower in mice colonized with microbiota from SEAN patients than in those colonized with microbiota from the other two donor groups, i.e., acute AN and HCs. This was most likely due to insufficient transfer of the microbial producers responsible for generating these two SCFAs. During induction of the ABA phenotype, the most pronounced alterations were observed in butyric acid abundance. Its levels decreased in all FMT groups of ABA mice (Fig. 5B). This reduction appears to be driven by the combination of food restriction and activity (ABA), since neither factor alone produced a comparable decrease in butyric acid levels (Fig. S5). Besides serving as an important energy source for colonocytes and hepatocytes, butyrate can enter the systemic circulation, cross the blood-brain barrier, and affect signaling within the central nervous system. Among other effects, it contributes to the regulation of appetite and circadian rhythms^40^. As discussed below, we identified both dysregulation of central orexigenic signaling and impaired circadian rhythms in ABA mice. Butyric acid can also modulate enteric serotonin synthesis in enterochromaffin cells^41^.

In our study, we observed a significant decrease in butyric acid levels in “AN-like” (ABA) mice compared to control mice fed *ad libitum* (AL) during the induction of the ABA phenotype. This decrease was seen in mice colonized with microbiota from both healthy individuals and AN patients. At the same time, tryptophan and serotonin levels were higher in ABA mice than in AL mice after the ABA induction phase (Fig. 6). Although the mechanisms by which enteric serotonin affects serotonergic signaling in the central nervous system are not fully understood, substantial evidence indicates disturbances in central serotonergic neurotransmission in patients with anorexia nervosa, which contribute to impaired homeostatic appetite regulation^42,43^. In contrast to serotonin, GABA levels were significantly reduced in SEAN-microbiota-colonized ABA mice compared with control mice. GABA is an essential inhibitory neurotransmitter in the CNS, and increasing evidence suggests that peripheral GABA signaling may influence brain function^44^. We can speculate on its indirect effect on AN-related behavior, since it participates in both homeostatic appetite (by inhibiting appetite-stimulating neurons) and hedonic appetite regulation (by inhibiting dopaminergic signaling)^45,46^. Moreover, the GABAergic system in the hippocampus and prefrontal cortex appears to modulate resilience or vulnerability to anorexia nervosa^47^. The levels of neurotransmitters in the gut can also influence the growth of specific bacteria, altering the composition and diversity of the gut microbiome^5^. These changes may in turn contribute to microbial dysbiosis and altered signaling along the microbiome–gut–brain axis.

Unfortunately, we were unable to perform a reliable analysis of associations between microbial taxa and concentrations of individual SCFAs and neurotransmitters. The relatively small number of mice within each experimental subgroup, defined by donor microbiota and differential access to food and running wheels, limited the statistical power needed to detect such relationships.

In our experimental setup, we observed behavioral differences in food intake and running activity in mice colonized with different microbiota even during the baseline phase of the experiment (Fig. 1). Notably, mice with microbiota transferred from patients with acute AN showed reduced food intake, whereas those with SEAN donor microbiota exhibited increased running activity (Fig. 7). However, when we introduced the ABA phenotype, its development proceeded similarly across all FMT groups. In the context of anorexia nervosa, this suggests that the gut microbiota may not play a major role in the onset of the disorder; nevertheless, it may contribute to disease progression. Interestingly, despite their reduced food intake, mice colonized with acute AN microbiota exhibited higher body weight compared to those colonized with microbiota from healthy controls. Similar findings, i.e., reduced food intake but comparable weight gain in mice transplanted with AN-derived microbiota, have been reported elsewhere^39,48^. This pattern suggests that the AN-associated microbiota may be adapted to extract maximum energy from low-calorie diets, making the energy available not only to microorganisms but also to the host.

Interestingly, we observed altered diurnal activity in ABA mice colonized with distinct microbiomes (Fig. 8). Mice transplanted with SEAN microbiota had the highest activity early in the morning and the lowest during dawn. This pattern contrasts not only with the two other FMT groups but also with the typical behavior of *Mus musculus*, a nocturnal species typically most active during dusk and dawn. Dopamine is a neurotransmitter that links circadian rhythms, the reward processing system, and vulnerability to addictive-like behaviors^49^. Altered dopamine signaling in AN patients is frequently discussed in the literature; although both increased and decreased dopamine levels have been reported^50^. Our data from ABA mice support the hypothesis that dopamine changes evolve progressively during the development of AN^51^, with the microbiome emerging as an important contributor to maladaptive dopaminergic signaling.

In our previous report, we showed alterations in homeostatic appetite regulation among germ-free, ATB-treated, and conventional ABA mice^14^. In the present study, we extend these findings and further confirm the importance of the gut microbiota in shaping feeding behavior, which is consistent with other reports^8,25,52^. Interestingly, we found significantly lower central hunger signaling, mainly through NPY and AgRP neuropeptides, in ABA mice transplanted with acute AN microbiota compared with the other two FMT groups of ABA mice (Fig. 9). These results correspond with the reduced food intake observed in mice colonized with acute AN during the baseline phase of the experiment (Fig. 7). Hunger and satiety perception are known to be dysregulated in patients with AN^9,53,54^ and this dysregulation appears to persist even during hospitalization^55^. Consistent with this, we previously showed that therapeutic nourishment during hospitalization did not substantially alter gut microbiome composition, SCFA profiles, or neurotransmitter levels^23^. Together, these findings suggest that improving the gut microbiome may be essential for achieving a full recovery from anorexia nervosa.

Dopamine release in the dorsal striatum, which consists of the caudate nucleus and the putamen (i.e., the caudate putamen), is essential for hedonic appetite regulation^56,57^. Consumption of palatable food is typically associated with dopamine release in this region. As discussed above, dopaminergic signaling is dysregulated in patients with AN^50^. In the induced ABA phenotype, we found significantly lower levels of the dopamine precursor tyrosine in the caudate putamen of ABA mice compared to all three control groups (i.e., mice fed *ad libitum* with or without a running wheel and mice subjected to food restriction; Fig. 10). Additionally, one of the dopamine biodegradation byproducts, 3-MT, was substantially decreased in ABA mice.

Besides the hedonic appetite regulation, the dopaminergic pathway is also involved in the reward response to physical activity. In patients with AN, restlessness, drive for movement, and even physical hyperactivity are frequent features of the disorder. Hyperactivity is also commonly associated with some of the psychiatric comorbidities of AN, such as anxiety, depression, and obsessive-compulsive disorder. Further, pathological exercise behavior in AN patients is associated with poorer outcomes and contributes to the chronification of the disease^58^. The transfer of microbiota from patients with AN into mice suggests that the gut microbiota may influence both appetite dysregulation and excessive activity in the ABA phenotype. This supports the idea that microbiome-dependent modulation of dopaminergic pathways could contribute to the behavioral phenotype characteristic of AN.

Our findings indicate that gut microbiota dysbiosis in patients with AN may contribute to disease progression and chronification, potentially through disruption of appetite-regulatory mechanisms. In translational model, we observed distinct behavioral and neurobiological phenotypes in mice following transplantation of microbiota derived from acute and SEAN patients. This supports the hypothesis that fecal microbiota transplantation from healthy donors could serve as a beneficial adjunctive intervention in the treatment of AN. Several human studies have reported the effects of FMT on gut microbiota^59–61^. Generally, both oral and rectal administration of microbiota from healthy donors improved the diversity and composition of the microbiome in patients with AN who underwent FMT in both the short-^60^ and long-term follow-up^59,61^ periods. However, improvements in psychopathology and appetite-related biomarkers appear less evident. Specifically, in a patient with SEAN, FMT was associated with sustained increase in bacterial diversity and improved barrier function over a one-year period, yet no concomitant changes in psychological outcomes were detected during follow-up^61^. In contrast, a patient with acute AN exhibited weight gain following FMT, which was maintained over a 36-week observation period^59^. Overall, these findings suggest that FMT may provide greater therapeutic benefit during the acute phase of the disease, when dysregulation of gut-brain axis signaling is not yet advanced. Given the resilience of the gut microbiome, we can speculate that a higher number of FMT treatment doses may be beneficial, as observed in other psychiatric conditions such as depression^62^. Modulation of the gut microbiome could represent a strategy to reduce relapse risk in AN, which may, in part, be driven by persistent microbiome dysbiosis.

This study is unique in distinguishing the effects of gut microbiota transferred into mice from patients in the early acute stage of anorexia nervosa and those with chronic severe and enduring anorexia nervosa. It is also the first study to investigate how distinct gut microbiota influence the development of activity-based anorexia in mice. Additionally, we provide the first characterization of fecal SCFA and neurotransmitter levels in ABA mice colonized with microbiota from AN patients and healthy controls.

Our study includes robust microbiome bioinformatic analyses; however, the small number of mice per experimental group limited our ability to perform more in-depth intercorrelation analyses to explore associations between gut microbiota and microbial metabolites.

Another limitation is the use of 16S rRNA gene sequencing, which lacks strain-level and functional resolution. This approach restricts the mechanistic interpretation of microbiota-driven effects in recipient mice and prevents detailed characterization of microbial pathways potentially involved in anorexia-related phenotypes.

A further potential limitation is the use of antibiotic-treated mice instead of germ-free (GF) mice, which have no interference with the original microbiome. However, GF mice represent an artificial model, and their use cannot be fully translated to human studies.

To conclude, our study is the first to clearly demonstrate distinct behavioral and physiological effects in mice after gut microbiota transfer from healthy controls, patients with acute AN, or patients with severe and enduring AN. Our findings suggest that these effects are primarily mediated by the short-chain fatty acid butyrate, as well as the neurotransmitters serotonin and dopamine. Contrary to our initial hypothesis, induction of the activity-based anorexia (ABA) phenotype did not differ among the FMT groups. Nevertheless, our findings support the hypothesis that AN pathophysiology develops progressively, with cumulative contributions from the gut microbiome. Early dysregulation of homeostatic appetite regulation may contribute to disease onset and progression, followed by impairments in dopaminergic signaling at later stages that may underlie disease chronification.

## Supplementary information

Supplementary Data

## Data Availability

Sequencing data are archived in the European Nucleotide Archive under project ERA35122707. Accession numbers with metadata for each sample, along with all R scripts used in the analysis, are available in the GitHub repository (https://github.com/JanetJezkova/Fecal-microbiota-transfer-from-anorexic-patients-into-ATB-treated-mice). The data from this study are available in the Open Research Repository Zenodo: 10.5281/zenodo.18244976.
