# Supplementary material for: Gut microbiota contributions to anorexia nervosa pathogenesis: insights from the activity-based anorexia model
Source: NPJ Biofilms Microbiomes. 2026 Jun 23;12:197. doi: 10.1038/s41522-026-01055-y (PMC13650143; doi:10.1038/s41522-026-01055-y)
Supplement: Supplementary file 1 [file 41522_2026_1055_MOESM1_ESM.docx]

**Supplementary data**

**Gut Microbiota Contributions to Anorexia Nervosa Pathogenesis: Insights from the Activity-Based Anorexia Model**

Radka Roubalová^1^*, Janet Luthar^1,2^, Petra Procházková^1^, Kateřina Zadáková^1^, Kristýna Coufalová^1^, Jakub Kreisinger^3^, Jaroslav Semerád^1,4^, Alena Nehasilová^1,4^, Hynek Mácha^1^, Dominika Luptáková^1^, Helena Tlaskalová-Hogenová^1^, Petra Holanová^5^, Alena Lambertová^5^, Hana Papežová^5^

*^1^Institute of Microbiology of the Czech Academy of Sciences, Vídenska 1083, Prague 4, Czech Republic*

^2^First Faculty of Medicine, Charles University, Katerinska 32, Prague 2, Czech Republic

^3^Faculty of Science, Department of Zoology, Charles University, Vinicna 7, Prague 2, Czech Republic

*^4^Institute for Environmental Studies, Faculty of Science, Charles University, Benatska 2, Prague 2, Czech Republic*

*^5^Department of Psychiatry, First Faculty of Medicine, Charles University and General University Hospital in Prague, Ke Karlovu 11, Prague 2, Czech Republic*

**Table S1**: Physical and clinical parameters and medication use of fecal microbiota donors. Differences in age, disease duration, body mass index (BMI), and body fat between groups were evaluated using the Kruskal–Wallis test followed by Dunn’s multiple comparisons test. Categorical variables were evaluated using the Chi-squared test. Results are shown as median with interquartile range (age, disease duration, BMI, body fat) or percentages (medication usage).

| **Variable** | **Acute AN (A) (n=10)** | **SEAN**  **(n=10)** | **Control (HC)**  **(n=10)** | **p-values** | | |
| --- | --- | --- | --- | --- | --- | --- |
|  |  |  |  | **A vs.**  **HC** | **SEAN vs. HC** | **A vs. SEAN** |
| **Age (years)** | 20 (20; 26.8) | 33.5 (28; 37) | 25.5 (24; 30.3) | NS | NS | **0.0021** |
| **Disease duration (months)** | 24 (18; 33) | 198 (133.5; 252) | NA | NA | NA | **<0.0001** |
| **BMI (kg/m^2^)** | 14.3 (13.7; 14.5) | 12.9 (11.7; 14.8) | 20.9 (20.1; 21.7) | **0.0018** | **<0.0001** | NS |
| **Body Fat (%)** | 3 (3; 3.33) | 3 (3; 3) | 25.7 (22.5; 27) | **0.0002** | **<0.0001** | NS |
| **Antidepressants (%)** | 60 (6/10) | 90 (9/10) | 0 (0/10) | **0.0147** | **0.00032** | NS |
| **Antipsychotics (%)** | 20 (2/10) | 50 (5/10) | 0 (0/10) | NS | **0.0389** | NS |
| **Anxiolytics (%)** | 10 (1/10) | 20 (2/10) | 0 (0/10) | NS | NS | NS |

**Fig. S1 Comparison between processed and fresh-frozen stool samples.** Differences in beta diversity (Bray-Curtis) between samples prepared for fecal microbiota transfer (FMT; n=10 in each donor group) and frozen stool samples (n=10 in each donor group). Statistical differences were assessed using PERMANOVA with the *adonis* function in R.

**Table S2:** Twenty most abundant donor genera transferred via FMT, shown as the number of ASVs grouped by genus (n_ASVs) and the relative abundance in the donor microbiome (rel_abundance %) for each FMT group.

**Table S3:** The LC-MS/MS parameters for analytical measurement of SCFAs

| **Compound** | **m/z of precursor ion** | **m/z of quantifier ion** | **m/z of qualifier ion** | **Polarity** | **Cell accelerator voltage [V]** |
| --- | --- | --- | --- | --- | --- |
| Acetic acid 3-NPH | 194.1 | 137.0 | 152.0 | negative | 4 |
| Propionic acid 3-NPH | 208.1 | 137.0 | 152.0 | negative | 4 |
| Butyric acid 3-NPH | 222.1 | 137.0 | 46.0 | negative | 4 |
| Isobutyric acid 3-NPH | 222.1 | 137.0 | 46.0 | negative | 4 |
| Isovaleric acid 3-NPH | 236.1 | 137.0 | 152.0 | negative | 4 |
|  | **Fragmentor [eV]** | **Quantifier collision energy [eV]** | **Qualifier collision energy [eV]** | **Retention time [min]** |  |
| Acetic acid 3-NPH | 120 | 20 | 16 | 2.3 |  |
| Propionic acid 3-NPH | 115 | 20 | 12 | 3.7 |  |
| Butyric acid 3-NPH | 120 | 20 | 44 | 6.0 |  |
| Isobutyric acid 3-NPH | 120 | 20 | 44 | 5.7 |  |
| Isovaleric acid 3-NPH | 125 | 20 | 16 | 7.7 |  |

**Table S4:** The LC-MS/MS parameters for neurotransmitter detection.

| **Compound** | **Precursor ion** | **Quantifier ion** | **Qualifier ion** | **Polarity** | **Cell accelerator voltage (V)** | **Fragmentor (eV)** | **Quantifier collision energy (eV)** | **Qualifier collision energy (eV)** | **Standard addition (ppb)** |
| --- | --- | --- | --- | --- | --- | --- | --- | --- | --- |
| **Dopamine** | 154.1 | 90.9 | 136.8 | pos | 4 | 72 | 26 | 10 | 50, 250, 1250 |
| **GABA** | 104.1 | 45.2 | 87.1 | pos | 4 | 50 | 30 | 10 | 5, 25, 125 |
| **Hydroxy-tryptophan** | 221.1 | 204 | 162 | pos | 4 | 90 | 10 | 18 | 10, 50, 250 |
| **Kynurenine** | 209.2 | 94.1 | 146 | pos | 4 | 70 | 14 | 26 | 10, 50, 250 |
| **Serotonin** | 177.1 | 160 | 115 | pos | 4 | 50 | 10 | 34 | 50, 250, 1250 |

*GABA = gamma aminobutyric acid; pos = positive.*

**Table S5:** List of primers used for gene expression analysis in the mouse hypothalamus

| **Gene** | **Forward Primer (5´→ 3´)** | **Reverse Primer (3´→ 5´)** | **Amplicon length (bp)** |
| --- | --- | --- | --- |
| *Hprt* | GGTTAAGCAGTACAGCCCCA | GGCCTGTATCCAACACTTCG | 81 |
| *Actb* | TTGCTGACAGGATGCAGAAG | GTACTTGCGCTCAGGAGGAG | 86 |
| *NPY* | CTGCGACACTACATCAATCT | CTTCAAGCCTTGTTCTGG | 124 |
| *AgRP* | TGCAGACCGAGCAGAAGAAG | GACTCGTGCAGCCTTACACA | 114 |
| *MCH* | AGCATCAAACTAAGGATGGCA | GGCGACAACGGATCTTTCTG | 195 |
| *Orexin* | ACCGTAACTACCACCGCTTT | GGGGGAAGTTTGGATCAGGA | 86 |
| *POMC* | CCATAGATGTGTGGAGCTGGTG | CATCTCCGTTGCCAGGAAACAC | 365 |
| *CART* | GCTGCTACTGCTACCTTTGC | TTCGATCAGCTCCTTCTCGT | 118 |

**Fig. S2**: Changes in beta diversity during the experiment. A – Differences in beta diversity across all sampling points; **S**ampling 1 (n=120), Sampling 2 (n=119), Sampling 3 (n=119), Sampling 4 (n=117). B – Differences in beta diversity among FMT groups at the post-FMT time point (sampling point 3); ctrl n=40, acutAN n=40, SEAN n= 39. Statistical differences were first assessed using PERMANOVA with the *adonis* function. To identify which specific group pairs contributed to the overall effect, pairwise PERMANOVA was performed using the *pairwise.adonis2* function.

| **FMT Group** | **% of transferred ASVs** | **% of total abundance** |
| --- | --- | --- |
| **FMT_ctrl** | 4.81 | 28.4 |
| **FMT_acute AN** | 6.09 | 72 |
| **FMT_SEAN** | 6.27 | 53.6 |

**Table S6**: The number of successfully transferred donor-derived ASVs and the proportion of the donor community represented by these transferred ASVs.

**Fig. S3**: Log_2_ fold changes for microbial taxa whose temporal variation was significantly affected by dietary restriction, irrespective of fecal transplant type (significant time point × dietary restriction interaction in LinDA). Group differences were assessed using Wilcoxon tests.

**Fig. S4**: Log_2_ fold changes for microbial taxa whose temporal variation was significantly affected by activity, irrespective of fecal transplant type (significant time point × activity interaction in LinDA). Group differences were assessed using the Wilcoxon test.

**Fig. S5**: Levels of SCFAs in mice during the activity-based anorexia induction phase. A – Levels of butyric acid in mice transplanted with microbiota from healthy controls and acute AN patients. B – Fold change in SCFA levels across mouse groups during the ABA phase. Group differences were assessed using the Kruskal-Wallis test followed by Dunn´s multiple-comparisons test. **p < 0.01.***p < 0.001. AL = food *ad libitum*, FR = food restriction

**Fig. S6**: Association between daily running distance and body weight in mice transplanted with microbiota from healthy controls (A), patients with acute AN (B), and chronic AN (**C**). r = Spearman correlation coefficient; FMT = fecal microbiota transfer; AN = anorexia nervosa; SEAN = severe and enduring AN.

**Fig. S7:** Daily activity during the ABA phase in mice transplanted with the microbiota from healthy controls (A **-** FMT_ctrl), patients with acute AN (B **-** FMT_acute), and chronic AN (C **-** FMT_SEAN). AL = food ad libitum; FR = food restriction (ABA mice).

**Fig. S8**: Expression of the orexigenic genes *NPY*, *AgRP*, and *orexin* among mice with different microbiota, shown in relation to their access to food and a running wheel. Differences were assessed using two-way ANOVA followed by Tukey´s multiple-comparisons test. AL = mice fed *ad libitum*; FR = mice with food restriction; AL, wheel = mice fed *ad libitum* with running wheel; FR, wheel = mice with food restriction with running wheel access.
